# Systematic Evaluation of the Effects of Voluntary Activation on Lower Extremity Motor Thresholds

**DOI:** 10.3390/jcm12185993

**Published:** 2023-09-15

**Authors:** Jasmine J. Cash, Mark G. Bowden, Andrea D. Boan, Lisa M. McTeague, John H. Kindred

**Affiliations:** 1 Department of Health Sciences and Research, Medical University of South Carolina, Charleston, SC 29425, USA; kindred@musc.edu; 2 Department of Clinical Integration and Research, Brooks Rehabilitation, Jacksonville, FL 32216, USA; mark.bowden@brooksrehab.org; 3 Department of Pediatrics, Medical University of South Carolina, Charleston, SC 29425, USA; boan@musc.edu; 4 Department of Psychiatry and Behavioral Sciences, Medical University of South Carolina, Charleston, SC 29425, USA; mcteague@musc.edu; 5 Ralph H Johnson VA Health Care System, Charleston, SC 29401, USA

**Keywords:** stroke, transcranial magnetic stimulation, resting motor threshold, active motor threshold

## Abstract

The purpose of this investigation was to elucidate the relationship between the resting motor threshold (rMT) and active motor threshold (aMT). A cross-sectional comparison of MTs measured at four states of lower extremity muscle activation was conducted: resting, 5% maximal voluntary contraction (MVC), 10%MVC, and standing. MTs were measured at the tibialis anterior in the ipsilesional and contralesional limbs in participants in the chronic phase (>6 months) of stroke (*n* = 11) and in the dominant limb of healthy controls (*n* = 11). To compare across activation levels, the responses were standardized using averaged peak-to-peak background electromyography (EMG) activity measured at 10%MVC + 2SD for each participant, in addition to the traditional 0.05 mV criterion for rMT (rMT_50_). In all participants, as muscle activation increased, the least square mean estimates of MTs decreased (contralesional: *p* = 0.008; ipsilesional: *p* = 0.0015, healthy dominant: *p* < 0.0001). In healthy controls, rMT_50_ was significantly different from all other MTs (*p* < 0.0344), while in stroke, there were no differences in either limb (*p* > 0.10). This investigation highlights the relationship between rMT and aMTs, which is important as many stroke survivors do not present with an rMT, necessitating the use of an aMT. Future works may consider the use of the standardized criterion that accounted for background EMG activity across activation levels.

## 1. Introduction

Repetitive transcranial magnetic stimulation (rTMS) is a method of non-invasive brain stimulation that is safe, effective, and Food and Drug Administration (FDA)-approved for the treatment of refractory major depression, smoking cessation, and obsessive compulsive disorder. The effects of rTMS are based on the magnetic field generated by a coil placed over the area of the cortex of interest, which ultimately induces repeated trans-synaptic depolarization [1], leading to improvements in behavioral and neurophysiological outcomes. In addition to neurological and psychiatric conditions [2,3], several investigations have explored the use of rTMS as an adjuvant to neurorehabilitation, particularly motor rehabilitation in stroke [4,5]. Currently, findings are mixed, in part due to the heterogeneity of procedures used across studies, as well as the lack of reproducible results [6].

To achieve the necessary stimulation power to facilitate or depress neural excitability, the International Federation of Clinical Neurophysiology (IFCN) practical guidelines suggest dosing rTMS based on some percentage of the motor threshold [7,8]. The motor threshold, or the percentage of maximal stimulator output (%mso) needed to elicit a response, is traditionally performed in a resting state, termed the resting motor threshold (rMT). The resultant response is evidenced by a twitch of the targeted muscle, called a motor-evoked potential (MEP), measured using surface electromyography (sEMG). 

Despite the potential of its utility in the neurorehabilitation field, many stroke survivors do not present with a rMT in their contralesional and/or ipsilesional limbs [9]. To ameliorate this, investigators commonly seek a motor threshold measured during voluntary activation of muscle, termed an active motor threshold (aMT). Utilizing this technique, an MEP is more probable at a lower stimulator power due to the increased corticospinal excitability secondary to activation of the muscle and reduced spinal inhibition. Differing amounts of background activation (and its non-standardized effect on motor threshold) make it difficult to compare dosing parameters and subsequent effects across investigations. It is unknown if the determination of stimulus intensity in a resting condition is appropriate for conducting measures in active conditions, and vice versa. To accurately dose rTMS to a level likely to enhance the neuroplastic state of the tissue and maintain safety, establishing the association between rMT and aMT is warranted.

Comparing rMT and aMTs across investigations is often hampered by the criterion utilized to determine a response. A rMT is traditionally determined by the amount of stimulator power needed to produce a MEP indexed with sEMG, with a peak-to-peak amplitude greater than 0.05 mV in more than 50% of stimulations. Currently, the selection of the nature of the aMT (i.e., the level of contraction) is arbitrary, and it is unknown how it relates to a comparable response in rested muscles. Additionally, there is no standard on how to measure aMT, namely because the criteria to determine an aMT are more variable. Traditionally, criteria of 0.1 mV to 0.2 mV are used to account for increased background sEMG activity but do not control for varying amounts of volitional activation [8]. The variable muscle activation paired with the differences in voltage criteria gives rise to inconstant amounts of the motor pool being excited across protocols. Taken together, this renders it difficult to compare not only the amounts of stimulation required to elicit an MEP across activation states but also the responses across studies. It is currently difficult to surmise if heterogenous results in rTMS effectiveness could, in part, be due to either “under-dosing” or “over-dosing” based on the current variability of the criteria used to derive motor thresholds. For example, it may be that in dosing rTMS on some percentage of an aMT, we may be “under-dosing” individuals, as it will take less stimulator power to elicit an aMT. A systematic comparison is warranted to determine differences in motor thresholds in different activation states to ultimately determine optimal levels of rTMS dosing. 

A potential solution to ameliorate the differences in voltage criteria and muscle activation to study the relationship between rMT and aMTs is to use one criterion that accounts for background muscle activation across activation states. We developed a criterion that was individualized for each participant using the activation state that yielded the greatest background peak-to-peak activity. In this way, the criterion is standardized, in that it was derived in the same manner, but also individualized to each participant’s volitional background sEMG activity to allow for within-person comparisons of motor thresholds by activation state. The amount of muscle activation to generate the criterion is based on the percentage of maximal voluntary contraction (MVC), as opposed to a percentage of sEMG, to better reflect motor neuron recruitment at various activation levels. It may be that sEMG activity does not increase linearly in concert with force output [10], which would make it difficult to differentiate levels of activation based on sEMG activity. Additionally, it may be easier for post-stroke individuals to maintain a percentage of force output, as some may exhibit decreased levels of motor control. In using a single motor threshold criterion across activation states informed by MVC, we can better discern the stimulator power necessary to elicit an MEP across similar motor neuron recruitment levels. 

As a further complication regarding integrating rMT and aMT findings, motor threshold recommendations are primarily determined based on use in the upper extremities [8]. Lower extremity cortical muscle representations are deeper within the interhemispheric fissure and require a coil with a more diffuse magnetic field for excitation to occur, e.g., a double-cone coil. Lower extremity muscle activation also requires greater spinal integration, hence the need for continued investigations specifically into lower extremity TMS-derived responses [11,12,13]. To determine the relationship between rMT and aMT in the lower extremities, a need exists to establish a criterion that allows for the comparison of responses regardless of the level of muscle activation or background activity. Additionally, there must be considerations and procedures specific to exciting lower extremity cortical muscle representations, such as the use of the double-cone coil. 

The purpose of this investigation was to compare the necessary amount of TMS power required to elicit a response across several activation states in healthy control participants and those in the chronic phase (>6 months) of stroke. We developed a method for deriving a motor threshold in the lower extremities and across levels of activation using individualized voltage criteria that accounted for background sEMG activity. Our central hypothesis for this investigation was that using a criterion that accounted for background sEMG activity would provide insight into the relationship between motor thresholds across activation states. Our first aim was to demonstrate the utility of said criterion to assess the relationship between motor thresholds derived from various activation levels in the lower extremities. Our second aim was to demonstrate the relationship between the motor thresholds derived using the set criterion and the often-used 0.05 mV criterion for rMT. 

## 2. Materials and Methods

### 2.1. Design

This study employed a cross-sectional analysis of motor thresholds measured across four states of muscular activation: rested (rMT_Standard_), 5% of maximal voluntary contraction (MVC) (5%MT), 10% of MVC (10%MT), and while standing (sMT). These specific activation states were chosen both because they are commonly used to elicit active motor thresholds and because these lower levels of activation are more likely to be achievable than higher levels of activation in post-stroke individuals [14,15]. We additionally chose 5%MVC and 10%MVC to mitigate any confounding factors due to fatigue, as greater levels of contraction would decrease motor unit recruitment. Lastly, a standing condition was utilized because it activates descending motor circuits specific to standing posturing. Data were collected during a single visit. Measures were collected from the dominant tibialis anterior (TA) in non-neurologically impaired individuals (controls) and the contralesional and ipsilesional limbs in individuals post-stroke. 

### 2.2. Participants

Eleven non-neurologically impaired and eleven individuals in the chronic stage of stroke (ages 18–85 years) were recruited for this investigation. Informed consent was obtained prior to enrollment and data collection with the approval of the Institutional Review Board, and in accordance with the Declaration of Helsinki. Healthy control participants were included if they had no self-reported neurological diseases/disorders and had no orthopedic limitations adversely affecting a passive range of motion. Inclusion criteria for individuals post-stroke were as follows: >6 months post-stroke; able to ambulate at least 10 m (with or without assistance); have no self-reported concurrent neurological diseases/disorders (e.g., multiple sclerosis, spinal cord injury, etc.); no orthopedic limitations adversely affecting a passive range of motion; no stroke localized at the cerebellum or brainstem; and can understand and provide informed consent. All individuals with contraindications to TMS as previously outlined by Rossi et al., 2021, were excluded [16]. 

### 2.3. TMS Data Set-Up

**Figure 1:**
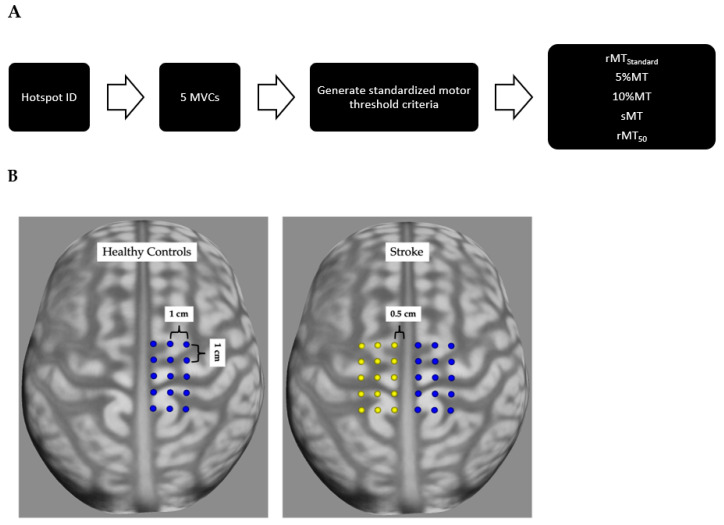
TMS data acquisition procedures. (**A**) Order of procedures: Using a 3 × 5 grid, we identified the motor hotspot. We then performed five maximal voluntary isometric contractions (MVCs). Using the averaged surface electromyography (sEMG) activity from 10% of the MVC, we calculated the standardized criterion. Lastly, using the hotspot and the standardized criterion, we derived each of the motor thresholds in a randomized order. (**B**) Averaged brain image from neuronavigational software, showing a 3 × 5 grid centered over the motor cortex. Only a single grid corresponding with the dominant limb was placed in healthy controls, and bilateral grids were placed in post-stroke individuals.

TMS data acquisition procedures are outlined in Figure 1. The skin over the TA was cleaned with alcohol-soaked pads. Disposable, single-use, bipolar (2 cm spacing), Ag/AgCl sEMG electrodes were placed over the dominant limb TA (healthy), and the contralesional and ipsilesional limb TAs (stroke). A reusable elastic sports bandage was wrapped around the electrodes and wires to reduce any movement artifact during testing and better secure the electrodes to the skin. The quality of the sEMG signals was tested, and deemed sufficient if there was <0.025 mV of sEMG activity with the participants’ muscles at rest. Once sEMG signals were of sufficient quality, the participants were registered to the neuro-navigation system (Brainsight, Rogue Research, CAN) using the standard MNI brain and head model native to the software. A 3 × 5 point grid, with 1 cm between each grid point and 0.5 cm lateral to the interhemispheric fissure, was placed over the dominant limb hemisphere in healthy control participants, and over both hemispheres in post-stroke individuals, before a participant’s arrival. 

### 2.4. Motor Threshold Criterion

**Figure 2:**
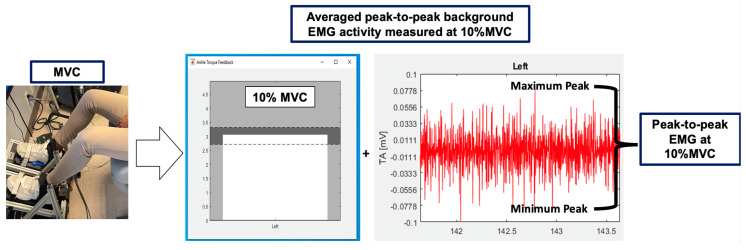
Motor threshold criterion. Participants performed maximal voluntary contractions (MVC) of the tibialis anterior, i.e., dorsiflexion. The torques were recorded using built-in force plates. The average of the MVCs was calculated and each percentage of the averaged MVCs was used and displayed to each participant (dark grey band). The white bar was reflective of each participant’s level of torque. When the white bar reached the dark grey band, the background peak-to-peak sEMG activity was recorded. The 10% of the MVC + 2SD was then added, and this value was used as the standardized motor threshold criterion.

Once prepared, participants then performed five isometric maximal voluntary contractions (MVC) of the TA, i.e., dorsiflexion, using a custom device that standardizes ankle position and measures ankle torque via built-in force plates under each foot (Figure 2). The recorded torque of the contractions was averaged using a custom-scripted code in MATLAB (The MathWorks, Natick, MA, USA). 

Based on pilot testing, the sEMG activity associated with 10% of the MVC elicited the greatest amount of background activity compared to the other conditions. The average of the background peak-to-peak sEMG activity + 2 standard deviations (SD) collected during a contraction at 10% MVC was used as the criterion to determine a motor threshold response across activation levels. This criterion allows for a clear MEP to be distinguished from the background sEMG activity produced during muscle contractions (Figure 2). To obtain this criterion, each participant was presented with visual feedback of a “target range” representative of 10% of their MVC (see dark grey band in Figure 2). Participants were asked to contract their muscles to this level while background sEMG activity was recorded. 

### 2.5. TMS Data Acquisition

TMS pulses were delivered via a double-cone coil (The Magstim Company Ltd., Carmarthenshire, UK) powered by two Magstim 200^2^ units connected via a BiStim Module operating in simultaneous discharge mode with the current traveling in an anterior–posterior direction. TMS pulses were applied over the same scalp location determined to produce the MEP with the greatest amplitude in the sitting position, or the “hotspot”. To determine the rMT_Standard_, participants were encouraged to relax their muscles while investigators used visual feedback to ensure there was no volitional sEMG activity. To determine the 5%MT and 10%MT, participants were asked to contract until they reached the “target range”, e.g., 5% of the MVC ±10%, or 10% of the MVC ±10%. The target range was visually provided to participants via a dark grey band (Figure 2). To determine the sMT, participants were asked to maintain a standing posture. Visual feedback was provided to ensure equal weight distribution on a dual-top force plate. Participants were connected to the ceiling with a harness during all standing assessments. The standing procedures were used by our research group and were previously detailed [17]. Simple adaptive parameter estimation by sequential testing (PEST) was used to determine motor thresholds at the various activation levels [18,19]. The individualized criteria were used across all activation states to identify motor thresholds. The testing order for the activation states was randomized for each participant. The traditional rMT using the 0.05 mV criterion, or rMT_50_ was also recorded. Unfiltered sEMG signals were amplified at 2000× (MA411 preamplifier electrodes, MA300-XVI Motion Labs Systems, Baton Rouge, LA, USA) and recorded at 5000 hz (1401 analog-digital converter, Cambridge Electronic Designs, Cambridge, UK).

### 2.6. Statistical Analysis

All analyses were conducted using SAS v9.4 (SAS Institute Inc., Cary, NC, USA). Three general linear mixed models with compound symmetry covariance structures were used to separately compare motor thresholds across all activation levels in the dominant (healthy controls), contralesional, and ipsilesional limbs (stroke). Repeated measures were accounted for in all models. If significant trends were observed in the full models, post hoc pairwise comparison assessments of least squares mean (LSM) estimates were performed. In secondary analyses, individual paired t-tests were used to explore comparisons between rMT_50_ arithmetic means and all other motor thresholds means, e.g., rMT_50_ and 10%MT, rMT_50_ and 5%MT, rMT_50_ and sMT, and rMT_50_ and rMT_Standard_. A significance level of *p* < 0.05 was adopted for all conditions.

## 3. Results

### 3.1. Participant Characteristics

Eleven post-stroke (*n* = 11, mean age = 60.9 (8.1), 7F/4M) and eleven healthy control participants (*n* = 11, mean age = 39.9 (9.4), 6F/5M) completed all study procedures. No individuals withdrew from the study. The MVC and subsequent individualized criteria were higher in healthy control participants than in either the contralesional or ipsilesional limb in post-stroke individuals (Table 1). 

### 3.2. Healthy Control Participants

**Figure 3:**
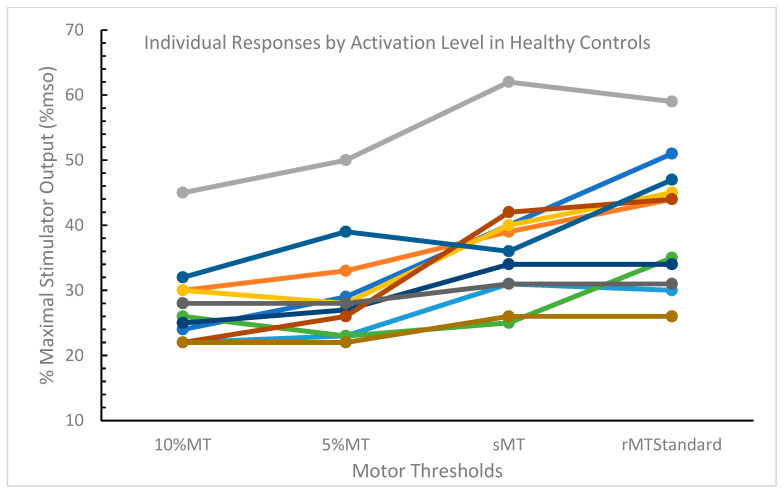
Individual responses by activation level in the dominant limb in healthy control participants. Each participant is represented by a different color. Abbreviations: MT, motor threshold; sMT, standing motor threshold; rMT_Standard_, resting motor threshold using the standardized criterion.

Motor thresholds were attained at all activation levels in healthy control participants (*n* = 55 motor thresholds). Motor threshold mean estimates were statistically different across activation levels using the standardized criterion (F = 23.89, *p* < 0.0001) (Table 2) and were not linearly associated with presumed muscle recruitment. Differences in LSM estimates demonstrated that the 10%MT, was significantly less than the sMT (*p* < 0.0001) and the rMT_Standard_ (*p* < 0.0001). Similarly, the 5%MT was significantly less than the sMT (*p* < 0.0001) and the rMT_Standard_ (*p* < 0.0001). The sMT was also significantly less than the rMT_Standard_ (*p* = 0.0436). Only the 10%MT and 5%MT estimates were not statistically different (*p* = 0.2558). Individual responses by activation level are presented in Figure 3.

Individual comparisons were conducted between the rMT_50_ mean (33.1 (7.9)%mso) and the other motor thresholds. We found that rMT_50_ was significantly less than rMT_Standard_ (*p* = 0.0039) and sMT (*p* = 0.0344), and significantly greater than 5%MT (*p* = 0.0068) and 10%MT (*p* = 0.0002). 

### 3.3. Post-Stroke Participants

**Figure 4:**
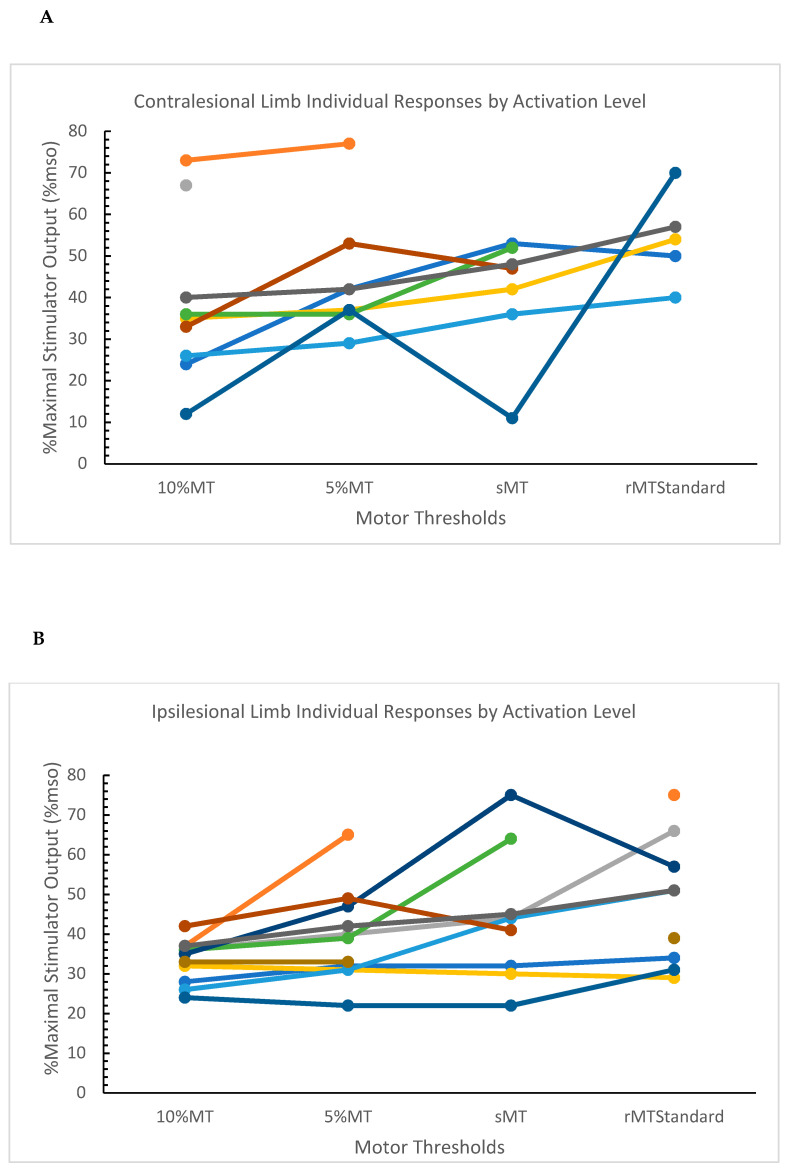
Individual responses by activation level in the contralesional (**A**) and ipsilesional limbs (**B**). Each participant is represented by a different color. Abbreviations: MT, motor threshold; sMT, standing motor threshold; rMT_Standard_, resting motor threshold using the standardized criterion.

In post-stroke participants, 87.3% (48/55) ipsilesional motor thresholds, and 61.8% (34/55) contralesional limb motor thresholds were attained. There was a main effect of activation level in the contralesional limb (F = 5.60; *p* = 0.008). Differences in LSM estimates demonstrate that the rMT_Standard_ was significantly greater than the 10%MT (*p* = 0.0008), 5%MT (*p* = 0.0149), and sMT (*p* = 0.0251). Additionally, we found a main effect of activation level in the ipsilesional limb (F = 6.82, *p* = 0.0015). Differences in LSM estimates demonstrate that the 10%MT was significantly less than rMT_Standard_ (*p* = 0.0003) and sMT (*p* = 0.0041). Additionally, 5%MT was significantly less than rMT_Standard_ (*p* = 0.0143) (Table 2). Individual responses by activation level are presented in Figure 4.

No significant differences were noted in the comparisons between the rMT_50_ arithmetic means of the contralesional (42.0 (7.9)) and ipsilesional (43.6 (12.9)) limbs and the motor thresholds derived using the standardized criteria.

## 4. Discussion

The purpose of this investigation was to compare motor thresholds derived while controlling for background sEMG activity across several muscle activation states in healthy controls and chronic stroke. We hypothesized that an individualized and standardized criterion would provide insight into the relationship between motor thresholds across activation states. We successfully determined the relationship between motor thresholds across activation states due to the use of a criterion that accounted for differences in background activity. In healthy control participants, we found an effect of muscle activation state on motor threshold using the standardized criterion. We additionally found that when comparing the %mso needed to generate a response using the standard 0.05 mV criteria to each of the motor thresholds using the standardized criterion, there were statistical differences found for each comparison. In post-stroke individuals, we found a main effect of muscle activation level on motor thresholds, with no main effect of side or interaction between activation level and side. Post hoc analyses using individual mixed models for each limb demonstrated that within each limb, there was a main effect of activation level. 

Given the potential utility of rTMS for use in neurorehabilitation, our preliminary findings offer an important first step in understanding a crucial dosing parameter in post-stroke individuals. Currently, the relationship between rMT and aMT, particularly in the lower limbs, is poorly understood. Much of our knowledge of the relationship between rMT and aMT is based on investigations in the upper limbs and a different model of motor control [20,21]. In line with the current investigation, it is routinely demonstrated that aMT measures are significantly lower than rMT due to the increased activation of the target muscle, which in turn increases corticospinal excitability [22,23,24]. Previous investigations in the upper extremities have also sought to describe the relationship between rMT and aMT [22,24,25]. The aMT of the first dorsal interosseous (FDI) muscle was found to correspond on average to 82% of rMT [22]. Additionally, practical guidelines give some indirect insight into the relationship between rMT and aMT, in that based on a stimulus–response curve, a stimulus intensity of 140% rMT or 170% of an aMT is suitable to excite muscles in the upper limbs [8]. While these guidelines do not provide a direct comparison of motor thresholds, they do provide insight into the comparability of the %mso needed to excite neural populations based on the nature of the motor threshold (i.e., resting vs. active). In using these recommendations and knowing the %mso of the test intensities, one can work backward to understand the relationship between motor thresholds derived at different activation levels. However, even in doing so, it may be difficult to extrapolate these results to the lower extremities. 

Typically, a double-cone coil is used to stimulate the cortical representation of the lower limb muscles. In comparison to the figure-of-eight coil (often used in upper limb studies), the double-cone coil generates a diffuse and more penetrating magnetic field, which in turn elicits higher intensities [16,26]. While an intensity of 170% aMT may be feasible for upper limb muscles and in healthy controls (where thresholds are lower), individuals with stroke tend to exhibit elevated motor thresholds for lower limb muscles. These higher intensities can not only potentially be uncomfortable for stroke patients and participants, but can also cause coactivation [27]. Although, even with higher stimulation intensities, it is not uncommon to be unsuccessful in eliciting a response in the rested musculature of the lower limbs in individuals post-stroke, as evidenced in this investigation. Conversely, the lower stimulus intensities that are derived from aMT, while more comfortable to participants and easier to elicit, may underestimate evoked responses [28]. Comparing rMT and aMTs directly allows for a better understanding of the necessary intensities to excite the target musculature to have a better idea of which intensities may be sufficient. To our knowledge, our preliminary findings are the first step to provide a necessary means of understanding the relationship between rMT and aMTs at commonly used levels of musculature activation in the lower limbs of post-stroke individuals. 

Traditionally, different criteria are used to derive the rMT (typically 0.05 mV) and the aMT (between 0.1 mV and 0.2 mV), without controlling the level of muscle activation, or background sEMG activity. The current investigation sought to compare motor threshold types by establishing one criterion across activation levels. In doing so, we determined the differences between the required stimulator output needed to excite a similar amount of the neural pool. The aMT criteria are typically greater to account for background sEMG activity; however, this can make direct comparisons more challenging because greater amounts of the neural pool are already excited. By setting the standard criterion to what we determined to be the “maximum” background activity for this investigation (i.e., 10%MVC + 2 SD), we were able to account for background activity while still allowing for a direct comparison regardless of activation level. 

We performed additional comparisons of all the resultant motor thresholds and the traditional rMT_50_ to indirectly compare potential stimulus intensities that would be derived from each of the motor thresholds. In healthy controls, we found the rMT_50_ was significantly different from all motor thresholds using the standardized criterion. Conversely, there were no differences between rMT_50_ and the motor thresholds derived using the standardized criterion that accounted for background sEMG activity in stroke. These findings are not surprising in neurologically healthy individuals, given that the motor neuron activation is not compromised in some way, which allows for greater levels of discernment between muscular activation regardless of the criteria used. In post-stroke individuals, the lack of differences highlights the potential to utilize criteria that are individualized to the participant, and can also allow for the use of motor thresholds in active musculature. As previously mentioned, rMTs can be difficult to obtain using the 0.05 mV criterion. If the rMT_50_ and 10%MT responses are similar, for example, we may be able to utilize the 10%MT for dosing, as we are more likely to see a response in the active musculature. However, whether this leads to “under-dosing” based on the use of an aMT versus an rMT remains to be seen. Despite the similarities in the %mso, future work may establish if rTMS effects on the neuroplastic state of the tissue are also similar. 

There were several limitations in the present investigation. First, there are a number of considerations when performing an investigation using TMS in post-stroke individuals. The number of participants in both the healthy control group (*n* = 11) and the post-stoke group (*n* = 11) are in line with similar investigations. However, given the nature of the population studied (i.e., stroke), it is common to either not obtain responses at all activation levels (namely resting), or when responses are obtained, stimulation intensity becomes uncomfortable for participants. These scenarios describe the reasons for missing data in the current investigation. To ameliorate this, we chose to utilize a general linear mixed model that would allow for comparisons of the data despite missingness. The model does assume data are missing at random, which was not the case in this investigation. However, using a mixed model is a more robust approach in accounting for missingness than other comparable methods of analysis that were considered. Future work necessitates a larger, and more vigorous assessment of the differences between rMT and aMTs to confirm our preliminary findings. Future work could also perform demographically matched comparisons of motor thresholds across activation states between healthy control participants and post-stroke participants. Second, our criterion of 10% MVC + 2 SD was selected based on our pilot testing. Specifically, 10% of the MVC was used because it was the activation level with the greatest background sEMG activity, and the + 2 SD, was used because participant responses would be representative of 95% of the data. We opted for the addition of 2 SD instead of the often-used 3 SD for two reasons: (1) the addition of 3 SD would make the threshold that much higher with the potential for making stimulation intensity uncomfortable, and (2) we believed there to be little difference in outlier responses captured at the top 2.5% (with 2 SD) of data versus the top 0.15% (with 3 SD). The use of a mean + 2 SD as a “threshold” is used across various other fields of study [28,29]. Third, we used the same “hotspot” to obtain all motor thresholds. It remains unclear whether the location of a motor map taken at rest and the location of a motor map taken during muscle contraction is similar. Fourth, while we did standardize weight distribution and foot position while obtaining the sMT responses, we did not standardize TA activation or force output in the same manner that was conducted for the other motor thresholds. Each participant’s TA activation during standing was mediated by strategies used to remain upright, therefore, particularly in stroke, this was difficult to standardize. Lastly, this work does not account for what effects the induced electrical field may have on motor threshold outcomes. The induced electrical field can differ widely across individuals, and therefore impact motor threshold outcomes. Such considerations should be integrated into future works. 

## 5. Conclusions

These preliminary findings offered two important contributions: (1) providing evidence as to the relationship between rMT and aMTs, and (2) the introduction of an individualized and standardized motor threshold criterion that can be used across activation states. Future studies can assess the comparability of neuromodulatory effects using percentages of the various motor thresholds.

## Figures and Tables

**Table 1:** Demographics of and characteristics of participants.

	Post-Stroke (*n* = 11)		Healthy Control (*n* = 11)
**Age,** µ (SD)	60.9 (8.1)		39.9 (9.4)
**Sex (F),** *n* (%)	7 (63.6)		6 (54.5)
**Stroke Hemisphere (L),** *n* (%)	7 (63.6)		
**Stroke Type (Ischemic),** *n* (%)	9 (81.8)		
**Walking Aid Use,** *n* (%)	2 (18.2)		
**Time Since Stroke (months),** *n* (%)	65.5 (28.7)		
**5-Times Sit to Stand (s),** *n* (%)	15.6 (6.6)		
**Limb, µ (SD)**	**Contralesional**	**Ipsilesional**	**Dominant**
**MVC (Nm)**	22.4 (9.6)	28.1 (12.2)	40.9 (50.1)
**Criterion (mV)**	0.2 (0.1)	0.3 (0.2)	0.4 (0.2)

Abbreviations: µ, mean; SD, standard deviation; F, female; L, left; S, seconds; Nm, newton meters; mV, millivolts.

**Table 2:** Motor threshold estimates (%mso).

	Healthy Control		Post-Stroke			
Limb	Dominant		Contralesional		Ipsilesional	
Motor Threshold	*n*	LSM (SE)	*n*	LSM (SE)	*n*	LSM (SE)
10%MT	11	27.8 (2.7)	9	38.4 (6.2)	11	33.3 (3.9)
5%MT	11	29.8 (2.7)	8	46.6 (6.4)	11	39.2 (3.9)
sMT	11	36.9 (2.7) ^a,b^	7	48.0 (6.6)	9	45.3 (4.1) ^a^
rMT_Standard_	11	40.6 (2.7) ^a,b,c^	5	63.0 (7.1) ^a,b,c^	9	49.2 (4.1) ^a,b^

Abbreviations: MT, motor threshold; sMT, standing motor threshold; rMT_Standard_, resting motor threshold using the standardized criterion; LSM, least squares mean; SE, standard error. ^a^ Significantly greater than 10% MT; ^b^ Significantly greater than 5% MT; ^c^ Significantly greater than sMT.

## Data Availability

Data from this investigation will be made available upon request. Please contact John Kindred, Ph.D., at kindred@musc.edu.
